# Fostering reproducible fMRI research

**DOI:** 10.1038/ncomms14748

**Published:** 2017-02-23

## Abstract

The validity of conclusions drawn from functional MRI research has been questioned for some time now. *Nature Neuroscience* and *Nature Communications* are committed to working with neuroimaging researchers to improve the robustness and reproducibility of their work.

Functional magnetic resonance imaging (fMRI) measures neural activity indirectly via the changes in the blood-oxygenation-level-dependent (BOLD) signal. It has been widely used by cognitive neuroscientists and psychologists to examine the neural correlates of higher cognitive functions in humans, such as decision-making, emotion regulation, social interactions and consciousness. Over the years, fMRI methods have become more refined, both in terms of the spatial and temporal resolution of imaging data and in terms of the statistical approaches used to analyse them. Researchers are no longer limited to making differential measurements of neural responses to various stimuli or task demands. Rather, current practices include decoding the information content from neural activations and using patterns of neural connectivity to predict an individual's cognitive abilities and traits.

Despite this remarkable progress, there are inherent challenges in fMRI studies. For example, we currently do not know the exact relationship between neuronal activity and the BOLD signal, making it hard to draw causal conclusions. Additionally, humans are highly variable in their task performance and their neural activity, as these can be influenced by mood, level of alertness, motivation, health and other factors. Finally, fMRI's dependence on image-processing pipelines and statistical analysis routines opens the door to any number of errors that can be introduced during the extraction of results. As a consequence, criticisms have been raised, suggesting that some fMRI findings are only modestly reproducible (Bennet, C. M. & Miller, M. B. *Ann. N. Y. Acad. Sci.* **1191**, 133–155 (2010)), and that some results could be interpreted as being overinflated or spurious (Eklund, A., Nichols, T. E. & Knutssen, H. *Proc. Natl. Acad. Sci. USA* **113**, 7900–7905 (2016)), incorrectly suggesting that a positive result is present (a ‘false positive'). Unfortunately, such reports have unintentionally harmed this technique's reputation and called into question the merit of published fMRI research. Are these criticisms warranted and, even if the answer is ‘no', how can the scientific community address the negative connotations associated with this research?

***Nature Neuroscience* and *Nature Communications* are committed to working with neuroimaging researchers to improve the robustness and reproducibility of their work.**

Even with the innumerable parameters that may differ between individual fMRI studies—study and task designs, scanner protocols, subject sampling, image preprocessing and analysis approaches, choice of statistical tests and thresholds, and correction for multiple comparisons, to name a few—many findings are reliably reproduced across labs. For example, the brain regions associated with valuation, affect regulation, motor control, sensory processing, cognitive control and decision-making show remarkable concordance across different fMRI studies in humans; these findings have also been supported by animal research drawing on more invasive and direct measures. These converging results should be highlighted in commentaries regarding research reproducibility, and critiques should be constructively balanced with potential solutions. In doing so, these critiques can provide an opportunity to revisit methods and highlight caveats, allowing the neuroimaging community to refine their methodological and analytical approaches and adopt practices that ultimately lead to more robust and reproducible results (http://www.ohbmbrainmappingblog.com/blog/keep-calm-and-scan-on).

One means of promoting reproducibility is to ensure transparent reporting of methodological details of study designs, data collection and analytical approaches, as well as any limitations to data interpretation. Papers sent out for peer review by journals within Nature Research include a completed methods reporting checklist and, upon acceptance, must comply with these reporting guidelines, which ensure that authors are clear about several aspects of experimental design and analyses (http://www.nature.com/neuro/journal/v16/n1/full/nn0113-1.html). To promote transparent methods reporting for MRI studies, we have developed an MRI-specific module to complement the methods reporting checklist. The aim is to capture essential MRI details that should be reported in every MRI-based research paper, and this module has been refined using suggestions recently provided by the neuroimaging community (https://doi.org/10.1101/054262). We hope these details will provide a clearer context within which our readers and reviewers can interpret MRI findings.

Beyond clearer methods reporting, reproducible science (Munafò, M. R. *et al*. *Nature Human Behaviour* **1**, 0021 (2017)) can also be fostered by increasing data accessibility. As part of Nature Research's growing efforts to support open science, primary research papers published in Nature journals now require mandatory statements about data accessibility (http://www.nature.com/news/announcement-where-are-the-data-1.20541; http://www.nature.com/authors/policies/availability.html#data). We also encourage researchers to deposit their data sets in recommended data repositories (http://www.nature.com/sdata/policies/repositories) so that they can be aggregated for large-scale analyses across studies, potentially improving the statistical power and robustness of any conclusions that may arise from these analyses.

*Nature Neuroscience* and *Nature Communications* recognize and applaud the unique advances obtained through fMRI research in cognitive neuroscience, psychology and human behaviour. As increasingly complex behavioural paradigms, analytical approaches and other noninvasive techniques are being implemented in fMRI-based research, we anticipate that this field will continue to evolve and grow. (*Nature Neuroscience* has assembled a Focus on Human Brain Mapping, highlighting exciting developments in fMRI and other noninvasive techniques: nn.4522). As with all fields of scientific research, technological developments and critical analyses of published literature are important means with which to improve methods, provide more robust and reproducible contributions and expand scientific knowledge. We look forward to working closely with our authors and peer reviewers to encourage, develop and publish the very best (and reproducible) fMRI studies.

Published online: 23 Feb 2017

